# Perianal Median Raphe Cyst: A Rare Lesion with Unusual Histology and Localization

**DOI:** 10.1155/2015/487814

**Published:** 2015-02-22

**Authors:** Betül Ünal, Cumhur İbrahim Başsorgun, Meryem İlkay Eren Karanis, Gülsüm Özlem Elpek

**Affiliations:** School of Medicine, Department of Pathology, Akdeniz University, 07070 Antalya, Turkey

## Abstract

Median raphe cysts present anywhere between the external urethral meatus and the anus. The cysts can occur at parameatus, glans penis, penile shaft, scrotum, or perineum. Perianal region is an extremely rare location for these lesions. Here we present a 50-year-old male patient who presented with a cystic, fluctuant lesion, located at 12 o'clock in perianal region. Microscopic examination revealed a cystic lesion with keratinized and nonkeratinized stratified squamous epithelium, pseudostratified ciliated epithelium, and scattered goblet cells. The final diagnosis of the lesion was median raphe cyst. Ciliated cells and perianal localization in median raphe cysts are extremely rare characteristics.

## 1. Introduction

Median raphe cysts are common benign lesions that present anywhere between the external urethral meatus and the anus, along midline [1]. It appears commonly in childhood or adolescents [2]. In most patients it is usually asymptomatic or unrecognized during childhood. The cysts become symptomatic with advancing age due to infection or trauma, which make diagnosis difficult. The cysts can occur at any site including parameatus, glans penis, penile shaft, scrotum, or perineum. Presentation of median raphe cyst in the perianal region is exceptional [2]. The epithelial lining of median raphe cysts includes urethral type, epidermoid type, glandular type, and mixed type epithelium [3]. Ciliated epithelium in median raphe cysts is an extremely rare finding [4–6].

According to our knowledge, in English literature our case represents the sixth case of median raphe cyst with ciliated epithelium. In addition, also this is the third case of ciliated median raphe cyst in the perianal region.

## 2. Case Presentation

**Figure 1:**
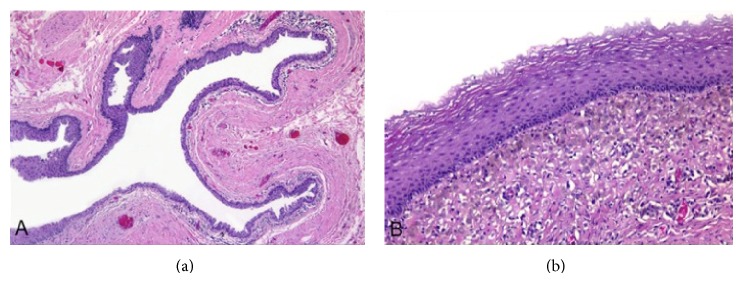
(a) Invaginated intradermal cyst. (b) Paved areas with stratified squamous epithelium (hematoxylin and eosin, magnifications (a) ×50, (b) ×200).

**Figure 2:**
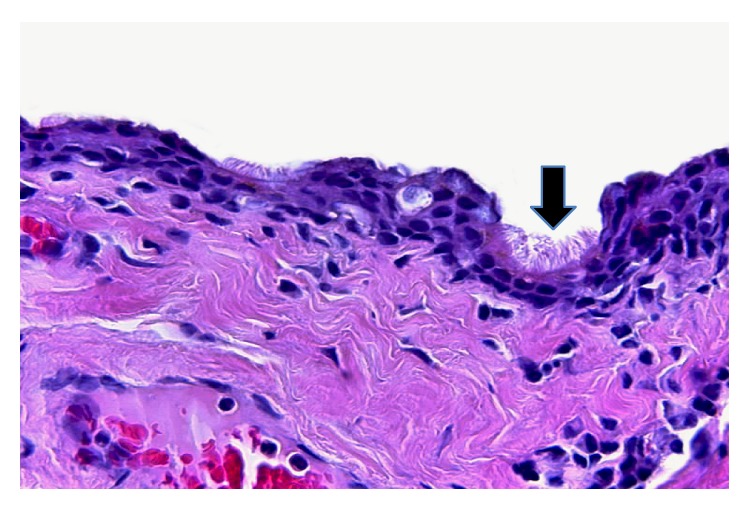
Cyst lining ciliated epithelium (arrow) (hematoxylin and eosin, magnification ×200).

**Figure 3:**
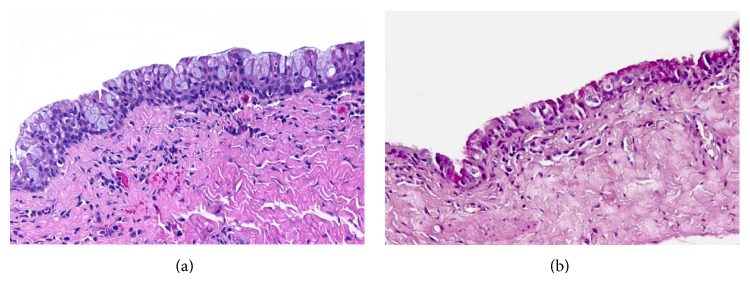
(a) Columnar epithelium with scattered goblet cells. (b) Bright pink mucin secretion in goblet cells ((a) hematoxylin and eosin and (b) mucicarmine, magnifications (a) and (b) ×200).

**Figure 4:**
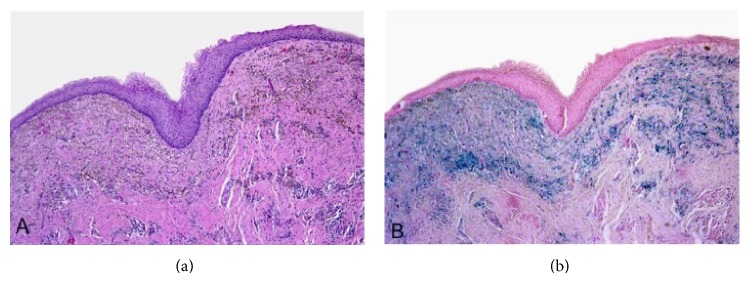
(a) Pigmented macrophages under stratified squamous epithelium. (b) This pigmented area consisted of blue colored hemosiderin and brown colored melanin ((a) hematoxylin and eosin and (b) Prussian blue, magnifications (a) and (b) ×100).

A 50-year-old male patient presented with a cystic, fluctuant lesion, located at 12 o'clock in perianal region. Any other pathology was not detected in perineal, scrotal, and perineal area. Surgical procedure was performed and cystic lesion was excised. Microscopic examination revealed a cystic lesion with a diameter of 37 mm and 3 mm wall thickness which was located in the dermis (Figure 1(a)). The epithelial lining of cyst consisted of keratinized and nonkeratinized stratified squamous epithelium (Figure 1(b)), pseudostratified ciliated epithelium (Figure 2), and scattered goblet cells (Figure 3(a)). Under the cyst epithelium, in some areas, hemosiderin laden macrophages, pigment of melanin, were seen. Immunohistochemical Melan-A and histochemical Prussian blue were performed for these areas (Figures 4(a) and 4(b)). Mucin secretion in goblet cells was shown by histochemical mucicarmine staining (Figure 3(b)). At 4 years of followup there was no evidence of recurrence.

## 3. Discussion

Median raphe cysts are rare congenital lesions along the male external genitalia. Until today, these cases were reported with different terms including mucoid cyst of the penile skin, genitoperineal cyst of the median raphe, parameatal cyst, hydrocystoma, and apocrine cystadenoma. It presents most commonly in the penile shaft [3, 5]. These types of cysts have been reported since 1910 [7] and such cases have been described in case reports. Shao et al. studied this issue in 55 patients with 56 median raphe cysts. According to their study, most (72.7%) of the patients were asymptomatic whereas 9 patients (16.4%) had infectious cysts. They stated that difficulty voiding in patients with cysts located on parameatus and distal prepuce. Respectively, cysts were found on the penile shaft, parameatus, prepuce, glans penis, and scrotum. In addition histopathological types of epithelium were urethral (55.4%), mixed (35.7%), epidermoid (5.4%), and glandular (3.4%) [3]. These findings support that our case is worthy of publication because of perianal location and presence of ciliated epithelium.

Ciliated cells in median raphe cysts are extremely rare and there were only 5 cases reported in the English literature (Table 1). Ciliated cells in median raphe cysts are probably a metaplastic change secondary to local irritation [3].

Median raphe cysts should be differentiated from other conditions such as epidermal cysts, pilonidal cysts, dermoid cysts, and urethral diverticula and especially for perianal location condyloma, viral wart, hemorrhoid, hypertrophied papilla, and neoplastic lesions [2, 8].

The pathogenesis of these cysts was not fully understood. Three different mechanisms have been described including fusion defect of urethral folds, development of the ectopic periurethral glands of Littre, and development from urethral columnar epithelium followed by separation [3].

Immunohistochemistry may be helpful for differential diagnosis in problematic cases.

The treatment of median raphe cysts is simple surgical excision and primary closure [9]. Median raphe cysts may regress spontaneously. Medical intervention may be required due to secondary infection and pain. Also small and asymptomatic cysts in infants can be observed without excision [10].

## Figures and Tables

**Table 1:** Summary of median raphe cysts with ciliated epithelium.

Literature	Age	Localization
Koga et al., 2007 [11]	27	Penis
Sagar et al., 2006 [5]	65	Perineum (perianal)
Fernández Aceñero and García-González, 2003 [6]	24	Penis
Scelwyn, 1996 [2]	62	Perineum (perianal)
Romani et al., 1995 [4]	N/A	Penis

N/A: not available.

## References

[1] LeVasseur J. G., Perry V. E. (1997). Perineal median raphe cyst. *Pediatric Dermatology*.

[2] Scelwyn M. (1996). Median raphe cyst of the perineum presenting as a perianal polyp. *Pathology*.

[3] Shao I. H., Chen T. D., Shao H. T., Chen H. W. (2012). Male median raphe cysts: serial retrospective analysis and histopathological classification. *Diagnostic Pathology*.

[4] Romani J., Barnadas M. A., Miralles J., Curell R., De Moragas J. M. (1995). Median raphe cyst of the penis with ciliated cells. *Journal of Cutaneous Pathology*.

[5] Sagar J., Sagar B., Patel A. F., Shak D. K. (2006). Ciliated median raphe cyst of perineum presenting as perianal polyp: a case report with immunohistochemical study, review of literature, and pathogenesis. *TheScientificWorldJournal*.

[6] Fernández Aceñero M. J., García-González J. (2003). Median raphe cyst with ciliated cells: report of a case. *The American Journal of Dermatopathology*.

[7] Otsuka T., Ueda Y., Terauchi M., Kinoshita Y. (1998). Median raphe (parameatal) cysts of the penis. *The Journal of Urology*.

[8] Nagore E., Sánchez-Motilla J. M., Febrer M. I., Aliaga A. (1998). Median raphe cysts of the penis: a report of five cases. *Pediatric Dermatology*.

[9] Soyer T., Karabulut A. A., Boybeyi Ö., Günal Y. D. (2013). Scrotal pearl is not always a sign of anorectal malformation: median raphe cyst. *Turkish Journal of Pediatrics*.

[10] Park C. O., Chun E. Y., Lee J. H. (2006). Median raphe cyst on the scrotum and perineum. *Journal of the American Academy of Dermatology*.

[11] Koga K., Yoshida Y., Koga M., Takeshita M., Nakayama J. (2007). Median raphe cyst with ciliated cells of the penis. *Acta Dermato-Venereologica*.

